# Progress in Ophthalmology

**Published:** 1896-11-21

**Authors:** 


### PROGRESS IN OPHTHALMOLOGY.

(*Continued from page* 114.)

At the same sitting the question of prolapse of iris in simple cataract extraction was also discussed, some members recommending suture of the corneal wound, others a conjunctural flap. Chibret mentioned that statistics varied from three to fifteen percentage of prolapse. We find in the Moorfields Hospital report that out of 1,517 cases there was a percentage of 13·86 of prolapse in "simple extractions," whilst in those with iridectomy only 0·87, a very significant fact. Dr. W. J. Collins gives us a very interesting paper on "The Pathology of Cataract" (*Medical Press and Circular*, July 8th, 1896). He gives tables showing his experiments on the composition of a number of freshly removed normal and cataractous lenses, in which he estimated four points—(1) The weight of the lens; (2) the total solids; (3) the amount of water; (4) the amount of ash. In the clear lenses the percentages of water and solids were 71 to 29, in the cataractous 64 to 36. He found that the cataractous lens is lighter and smaller than a clear one of the same age. He gives the following conclusions:—

"Cataractous lenses, as a general rule, contain relatively less water and more solids than non-cataractous lenses, and the dehydration, associated with opacification, is in no sense a change dependent upon age, and has no parallel in the ordinary changes which age brings with it in the clear lens."

Mr. Collins adds a very practical warning *apropos* of the difficulty in estimating the rate at which cataracts will mature, and quotes the old adage, "never prophesy till you know." He states that cataracts associated with myopia are usually slow in maturation as are also those beginning at the posterior pole. He also demonstrates the value of preliminary iridectomy as a means of hastening maturation.

Of recent suggestions in ophthalmic therapeutics, we may mention Dr. Allen Jameson's (*Brit. Journal of Dermatology*, April, 1896) new excipient for ointments. It is composed of ʒiii of lanoline to ʒss each of ol amygdalæ and distilled water. It is said to answer admirably as a simple ointment to prevent gluing of the lids, and will keep a long time with the addition of a little boric acid. It is non-irritating, and gives a comforting sensation of coolness.

Dr. Mackenzie Davidson (*The Therapist*, May 15th, 1896) reports most favourably on the value of formic aldehyde in corneal ulcers and abrasions, especially where there is septic infection. He uses a solution of formalin (formic aldehyde, 40 per cent. in water) 1 in 2,000 or 3,000 applied every hour. He states that the pain, so frequently a distressing symptom in hypopion ulcers, is speedily relieved, that it is non-poisonous, and non-irritating. Swan Burnet also advocates its use.

Brilliantof (*Les Nouvelles Remedies*, May 24th, 1896) records twenty cases in which he has with great advantage employed sub-conjunctoral injections of parachlorophenol for corneal ulceration, hypopion keratitis, iritis, and interstitial keratitis. The injections give rise to very little pain.

In the treatment of scleritis and episcleritis, Von Keuss (*The Medical Week*, June 5th, 1896), of Vienna, points out the value of electrical treatment by means of an uninterrupted current of 1 to 1½ milliamper. He applies it by means of a platinum electrode in immediate contact with the eyeball, which is first rendered anæsthetic by cocaine. The other pole is applied to the forehead, cheek, or hand. Sixty to ninety seconds every other day is recommended, and the effect is said to be rapid increase in the vascular injection at the point. The pain is said to be speedily relieved, and recovery may be expected in from ten to twelve sittings. The pain also in iritis, keratitis, and recent iridocyclitis is said to be greatly alleviated by this method.

Eucaine is coming more and more into use as a substitute for cocaine. Its advantages over the latter, according to Beyer (*Revue de Therapie*, June 15th, 1896), are, that it is not so toxic, it produces hyperæmia instead of anæmia, it does not cause desquamation of corneal epithelium, nor any paralysis of pupil or ciliary muscle.

As a substitute for iodoform, Hoor (*Brit. Med. Journ.*, July 18th, 1896) recommends nosophen, a compound of phenol and iodine. The substance, he states, is non-irritating, and can be used in corneal ulcers, burns, wounds, &c.

So far the Roentgen rays have been of little use to the ophthalmologist. Mr. Harnisch ("Annals of Ophthalmology-Otology," April, 1896) has demonstrated the comparative transparency of the different parts. The lens is the least transparent, lens capsule, choroid, and retina the most, whilst vitreous, cornea, and sclera occupy the intermediate position.

Dr. de Schweinitz (*American Journal of Med. Science*, June, 1896), of Philadelphia, in a recent treatise on Toxic Amyblopia, states that in New South Wales blindness is found in horses, the result of their eating the leaves of the Australian tobacco plant. Whilst tobacco, alcohol, lead, and quinine are the commonest toxic agents, he also quotes cases due to iodoform, nitro benzol, carbon bisulphide, arsenic, opium, chloral, potassium bromide, cannabis indica, caffein, thein, and the anæsthetics. In the treatment of quinine amblyopia he suggests pilocarpine injections, nitrite of amyl inhalations, and strychnine.

Some practical suggestions on direct transplantation of skin-grafts in the cure of entropion are made by Dr. Argyll Robertson (*The Practitioner*, August, 1896), who considers that the dangers of sloughing of the grafts are over-estimated. The following are the essential points to be attended to: "(1) Immobility of the eyelids; (2) the formation of a graft accurately fitting the raw surface to be covered; (3) the removal of all fat and cellular tissue from the graft; (4) the retention of the graft in apposition to the raw surface, with the least possible injury to or interference with the transplanted skin; and (5) the use of suitable antiseptic applications."

He recommends the inner surface of the arm as the best and most convenient source of supply. He secures immobility of the lids by stitching the lid edges together; the accurate measurement he assures by cutting out a paper pattern of the raw surface and cutting the skin graft from this, allowing one-fifth for shrinkage. The intimate application and safe retention of the flap he secures by means of "tethering" or "cradle" stitches; these are made with a continuous horsehair, which is introduced into the skin, not of the flap (which it never penetrates), but of the face close to the raw edge, near one extremity, and is passed across over the flap and through the skin on the opposite side, crossing and recrossing as may be found necessary. When oozing has ceased the surface of the graft and adjoining skin are washed with a gentle stream of corrosive sublimate solution (1 to 2,000), and then finely powdered iodoform dusted over. Then in turn come linen covered with iodoform ointment, corrosive sublimate lint, damp, and a pad of cotton wool. Both eyes are bandaged. He recommends that the dressings, unless for obvious reasons, should not be removed for forty-eight hours; they should be reapplied for six to eight days. The stitches may be removed on the fifth or sixth day.

A modification of Dr. Mules' (*International Med. Magazine*, May, 1896) ingenious operation for ptosis, is suggested by T. C. Evans (Kentucky). He makes his marginal lid incision much as Mules, but he makes a second incision down to the occipito-frontalis muscle, above the eyebrow, and passing his silver suture from below up, brings one along the line of the upper incision to the outer extremity, where they pass through a perforated shot; traction is made until the lift is sufficient and the shot squeezed with pliers and left in the wound. Both wounds are stitched, and these stitches removed in forty-eight hours.

Simeon Snell (*B. M. J.*, July, 1896) has employed electrolysis in the treatment of three cases of detached retina, in all these with resulting lessening of the detachment, and in one case with a large recovery of the field of vision.

